# Optimal Analysis Method for Dynamic Contrast-Enhanced Diffuse Optical Tomography

**DOI:** 10.1155/2011/426503

**Published:** 2011-07-31

**Authors:** Michael Ghijsen, Yuting Lin, Mitchell Hsing, Orhan Nalcioglu, Gultekin Gulsen

**Affiliations:** ^1^Department of Radiological Sciences, John Tu and Thomas Yuen Center for Functional Onco-Imaging, University of California, Irvine, CA 92697, USA; ^2^Department of Cogno-Mechatronics Engineering, Pusan National University, Pusan 609-735, Republic of Korea

## Abstract

Diffuse Optical Tomography (DOT) is an optical imaging modality that has various clinical applications. However, the spatial resolution and quantitative accuracy of DOT is poor due to strong photon scatting in biological tissue. Structural *a priori* information from another high spatial resolution imaging modality such as Magnetic Resonance Imaging (MRI) has been demonstrated to significantly improve DOT accuracy. In addition, a contrast agent can be used to obtain differential absorption images of the lesion by using dynamic contrast enhanced DOT (DCE-DOT). This produces a relative absorption map that consists of subtracting a reconstructed baseline image from reconstructed images in which optical contrast is included. In this study, we investigated and compared different reconstruction methods and analysis approaches for regular endogenous DOT and DCE-DOT with and without MR anatomical *a priori* information for arbitrarily-shaped objects. Our phantom and animal studies have shown that superior image quality and higher accuracy can be achieved using DCE-DOT together with MR structural *a priori* information. Hence, implementation of a combined MRI-DOT system to image ICG enhancement can potentially be a promising tool for breast cancer imaging.

## 1. Introduction

Diffuse optical tomography (DOT) utilizes near infrared (NIR) light to probe tissue optical properties, mainly absorption and scattering. DOT has shown promise in breast cancer characterization and neoadjuvant chemotherapy monitoring based on spatially resolved tissue intrinsic physiological parameters, such as total hemoglobin concentration, oxygen saturation, and water concentration. For example, total hemoglobin concentration of tumors was found to be two- to four-fold greater in tumors due to the enhanced tumor vasculature and blood supply. Meanwhile, the oxygen saturation levels were shown to be reduced compared to the normal tissue, due to the increased oxygen consumption by rapidly differentiating and metabolically active tumor cells [1–7]. Similarly, decreased total hemoglobin concentration is observed in breast cancer patients who responded to neoadjuvant chemotherapy treatment [1, 8–10]. 

Despite the success of preliminary clinical data, the inverse problem of DOT is severely ill-posed due to strong scattering of light by tissue, which leads to poor spatial resolution and low quantitative accuracy [11, 12]. Mainly, measurements on the boundary are not sensitive to the optical property variation induced by a small lesion embedded in a heterogeneous medium. Meanwhile, reconstructed absorption images are very sensitive to measurement noise, which results in significant artifacts caused by experimental uncertainties. Such uncertainties include optical probe coupling to the tissue surface, source-detector position uncertainty, and errors in determining the tissue boundary for proper modeling of light propagation. In addition to these problems, optical properties of the tissue differ significantly not only for different individuals but also for the same individual at a different physiological stage [6]. Furthermore, the widely used diffusion equation is only an approximated model to describe light propagation in tissue. This can cause modeling mismatch in the presence of a low scattering region, such as the bladder [13, 14]. To overcome these limitations, exogenous contrast agents can be used to obtain differential absorption images of the lesion using dynamic contrast-enhanced DOT (DCE-DOT) [15–18]. In particular, the contrast agent used in this study is Indocyanine green (ICG), an FDA-approved NIR absorber with peak absorption of 785 nm. By subtracting reconstructed baseline (precontrast) absorption images from the subsequent ones after the ICG injection (postcontrast), the relative absorption enhancement map is obtained. Both pre- and post-contrast measurements are acquired under the same conditions, and, hence, this method is only sensitive to the change of the optical signal. As a result, artifacts due to the modeling error, individual difference, and experimental uncertainty are remedied by this differential approach [15, 19]. In addition to the enhanced detection of lesions, the pharmacokinetics of ICG uptake can be a potential physiological marker for identifying breast lesion malignancy similar to the role of the MR exogenous contrast agent, Gd-DTPA, in dynamic contrast-enhanced magnetic resonance imaging (DCE-MRI). 

Another major problem of DOT is its low quantitative accuracy. Indeed, recovered optical properties for an inclusion highly depend on the depth and size of the lesion. This limitation cannot be overcome by using an exogenous contrast agent alone as demonstrated with phantom studies previously [18]. In order to improve the accuracy of DOT, structural *a priori* information from another high spatial resolution imaging modality, such as magnetic resonance imaging (MRI) or X-ray computed tomography (XCT), has been utilized to constrain and guide DOT reconstruction [7, 20].

There are only a few DCE-DOT studies using ICG as an exogenous contrast agent. Different analysis methods and systems are used for these studies. For instance, Cuccia et al. used diffuse optical spectroscopy (DOS) to obtain the ICG kinetics and showed different kinetics for different tumor types with an animal model [17]. Intes et al. and Ntziachristos et al. used continuous wave breast DOT systems and showed different kinetics for cancerous and normal breasts [15, 16]. Using ICG enhancement kinetic data obtained from eight patients, Rinneberg et al. observed that the absorption coefficient normalized to the tissue total hemoglobin concentration did not differ for the normal and cancerous breast tissue during the wash-out phase [21]. Following this study, Hagen et al. showed that invasive carcinomas retain ICG fluorescence signal after being largely cleared out from the body, indicating the high permeability of malignant tumors [22]. In all these studies, however, the number of cases was small and hence there was no conclusion about which reconstruction and analysis method was optimal for DCE-DOT. Furthermore, all of them were stand-alone DOT systems that did not utilize high resolution anatomical *a priori* information. In the last couple of years, our lab has developed a combined MRI-DOT small animal imaging system [23]. Initial results have demonstrated tumor enhancement using ICG [24]. In this study, we investigated and compared different reconstruction methods and analysis approaches for regular endogenous DOT and DCE-DOT with and without MR structural *a priori* information for arbitrarily shaped objects. We first compared the regular DOT and DCE-DOT results for various target-to-background concentrations. Eight realistic arbitrarily shaped phantoms were imaged and analyzed to show that the sensitivity of regular DOT results highly depends on the shape of the object, position of the inclusion, and the object to background contrast. However, the inclusion can be resolved in all eight cases even at the lowest contrast with contrast-enhanced DOT. In addition, we intended to look for the best DCE-DOT data analysis method in this study. We compared five different reconstruction methods and demonstrated that an accurate enhancement curve can only be obtained with MR *a priori* information. In addition to the phantom studies, an *in vivo* study was also carried out. Fisher rats bearing the R3230 breast tumor model were imaged by our combined DOT-MRI system. To be able to monitor the enhancement kinetics of ICG, time resolution of the DOT system has been increased to 16 seconds using a heterodyne detection technique [18, 24].

In summary, our phantom and animal studies have shown that superior image quality and higher accuracy can be achieved using DCE-DOT together with MR structural *a priori* information. Hence, implementation of a combined MRI-DOT system to image ICG enhancement can potentially be a promising tool for breast cancer imaging. 

## 2. Methods

### 2.1. Instrumentation

**Figure 1:**
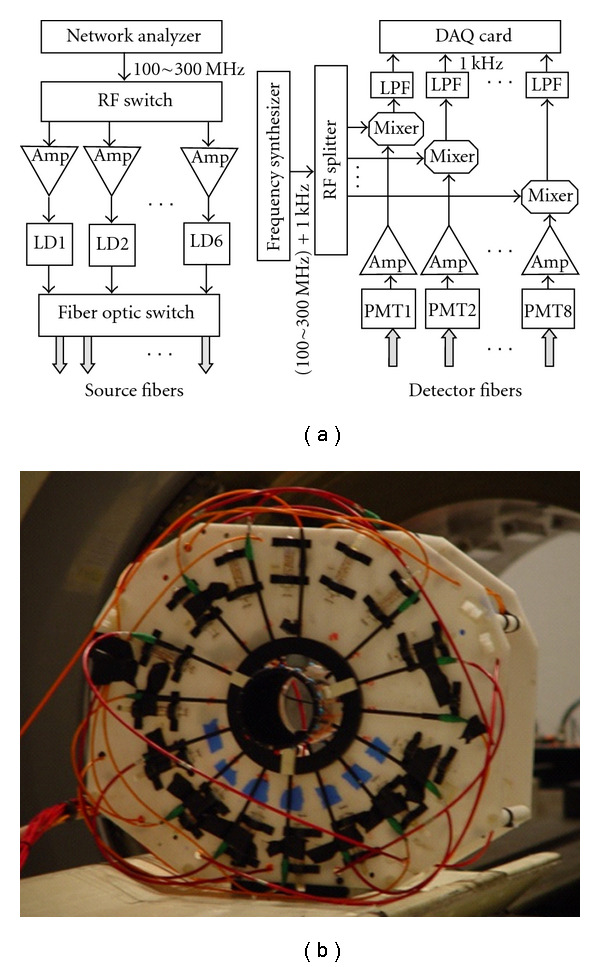
The hybrid MRI-DOT system. (a) Schematic of the DOT system. The system utilizes 6 laser diodes (LD 1–6). Only LD2 (785 nm) is used for DCE-DOT studies. The lasers are modulated at 100 MHz by the network analyzer. The optical signals are collected by detector fibers prior to transmission to the detection system, which uses PMTs as detectors. After downconverting to 1 kHz, signals are recorded simultaneously by a data acquisition card (DAQ). (b) The fiber optic interface. Eight sources and eight detectors are placed in fan-beam geometry. A 16-leg birdcage RF coil is integrated into the interface. When placed in the MRI bore, both optical and MR measurements can be acquired simultaneously.

A hybrid MRI-DOT animal imaging system has previously been developed in our lab. The schematic diagram and picture of the optic interface are shown in Figure 1. The system utilizes eight sources and eight detectors in a fan-beam geometry. The detector and source fibers that were chosen for this system are 1 mm step-index and 62.5 *μ*m gradient-index fibers, respectively. An 8 × 32 fiber optic switch (DiCon Fiberoptics, GP700) is used to multiplex the laser diode outputs through the source fibers. The lasers are modulated at 100 MHz, and photomultiplier tubes (Hamamatsu R7400-20) are used at the multiple detection sites. Output of each PMT is amplified by a 65 dB RF amplifier and recorded simultaneously using an NI 4472 8-channel DAQ system, after downconversion of the signal frequency to 1 kHz. Finally, the data is postprocessed to obtain 64-phase and 64-amplitude measurements. 

The MRI setup consists of a 4T MR system with a custom-designed birdcage type RF coil, which is built into the DOT interface. The MR console is developed by ISOL technology. Fiber optic cables are passed through a waveguide from the RF-shielded MRI room to the control room, where the DOT detection unit and data acquisition hardware are located. 

### 2.2. Forward and Inverse Models for Diffuse Optical Tomography

The diffusion equation is used to model the light propagation in tissue [12]. In the frequency domain, it can be written as

(1) $$\begin{array}{cc} & \nabla \cdot \left[D\left(\vec{r}\right)\nabla \Phi \left(\vec{r},\omega \right)\right]-\left[\mu_{a}\left(\vec{r}\right)+\frac{i\omega }{c_{n}}\right]\Phi \left(\vec{r},\omega \right)\\ & \;\;=-q_{0}\left(\vec{r},\omega \right), \end{array}$$

where the Φ(**r**, *ω*) is the fluence rate. *D*(**r**) (mm^−1^) denotes the diffusion coefficient, which is defined by *D* = 1/3(*μ*_a_ + *μ*_*s*_′). The reduced scattering and the absorption coefficients of the medium are represented as *μ*_*s*_′ (mm^−1^) and *μ*_*a*_ (mm^−1^), respectively. The modulation angular frequency and the speed of light in the tissue are represented by *ω* and *c*_*n*_, respectively. 

The Robin boundary condition relates the optical fluence rate to optical flux at the boundary and can be written as

(2) $$\Phi \left(\vec{r},\omega \right)+2AD\left(\vec{r}\right)\cdot \frac{\partial \Phi \left(\vec{r},\omega \right)}{\partial n}=0,$$

where *n* is the direction perpendicular to the boundary. *A* is the boundary mismatch parameter that accounts for the light reflection on the boundary surface, which is determined by Fresnel reflections [25]. Hence, the photon flux measurement on the boundary is

(3) $$\phi =-D\left(\vec{r}\right)\frac{\partial \Phi }{\partial n}=\frac{\Phi }{2A}.$$

A point source *q*_0_(**r**, *ω*) = *δ*(**r** − **r**_*s*_) is used in this simulation. The extrapolated boundary condition is applied to model the position of the source [25]. For each source position, eight measurements are obtained at the detector sites. Therefore, a total of 64-amplitude and 64-phase data are obtained from the forward solver. The diffusion equation is solved with the finite element method (FEM). The details have been described previously [26, 27]. 

The inverse problem is solved by minimizing the difference between the measured and calculated data according to the following error function:

(4) $$\varepsilon^{2}\left(\mu_{a}\right)=\overset{N_{s}}{\underset{i=1}{\sum }}\;\overset{N_{d}}{\underset{j=1}{\sum }}{\left(\phi_{ij}^{m}-P_{ij}\left(\mu_{a}\right)\right)}^{2}.$$

Here, *i* represents the number of sources and  *j*  represents the number of detectors. *ϕ*_*ij*_^*m*^ is the measurement. *P*_*ij*_(*μ*_*a*_) is the flux on the measured point calculated by the forward solver from the spatial distribution of *μ*_*a*_. We iteratively update the unknown *μ*_*a*_ with the Levenberg-Marquardt method by

(5) $$X_{m+1}=X_{m}+{\left(J^{T}J+\lambda I\right)}^{-1}\left(J^{T}\varepsilon \right),$$

where *ε*_*ij*_ = (*ϕ*_*ij*_^*m*^ − *P*_*ij*_) and *X*  represents the unknown matrix of *μ*_*a*_. The dimension of *X* is *N*. *N* represents the number of nodes in the FEM mesh. The Jacobian matrix *J* is calculated with the adjoint method [12]. 

When structural* a priori* information is utilized during the reconstruction process, “Laplacian-type *a priori*” developed by Yalavarthy et al. is implemented to find the absorption coefficient for the inclusion [28]. The *L*-matrix can be written as

(6) $$L_{ij}=\{\begin{array}{cc} 0 & \text{if}\;i\;\text{and}\;j\;\text{are}\;\text{not}\;\text{in}\;\text{the}\;\text{same}\;\text{region},\\ \frac{1}{N_{r}} & \text{if}\;i\;\text{and}\;j\;\text{are}\;\text{in}\;\text{the}\;\text{same}\;\text{region},\\ 1 & \text{if}\;i=j, \end{array}$$

where *N*_*r*_ represents the number of nodes included in one region. Then, the update equation can be expressed as

(7) $$X_{m+1}=X_{m}+{\left(J^{T}J+\lambda L^{T}L\right)}^{-1}\left(J^{T}\varepsilon \right).$$

When the source and detector coupling is considered, the coupling coefficient is explicitly recovered as unknowns from the measurements. This has been described by Boas et al. previously [29, 30]. The modified measurements are

(8) $$\Phi_{i,j}=s_{i}\times d_{j}\times \Phi_{i,j}^{\text{Forward}},$$

where *s*_*i*_ and *d*_*j*_ are the coupling factors for the *i*th source and *j*th detector position. 

For each arbitrarily shaped phantom, a finite element mesh is generated from the MR image. In order to reduce the computational cost, the dual mesh strategy is used. The following steps were followed during the mesh generation procedure.

**Figure 2:**
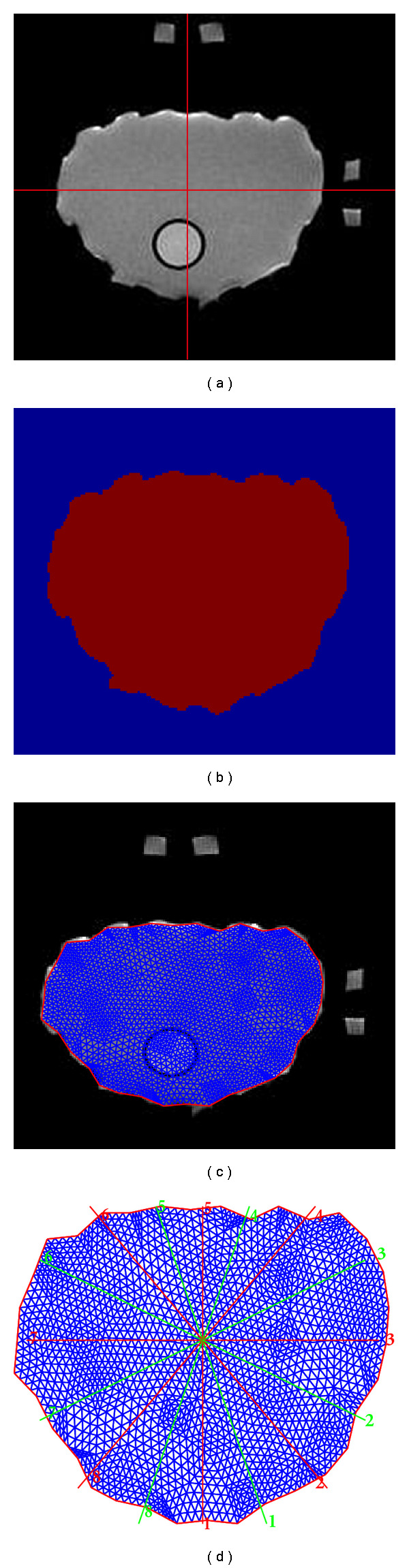
Finite element mesh generation from MR image. (a) Selection of the center points based on the fiducial markers. (b) A binary mask is generated representing the shape of the phantom from the MR image. (c) Finite element mesh is generated according to the exterior boundary obtained from the binary mask for optical data analysis. (d) Source and detector positions are then determined.

1. *MR and optical image coregistration*: the center of the RF coil and optical probes are determined from the MR image by using fiducial markers. As shown in Figure 2(a), the center is determined using two orthogonal markers manually.
2. *XCT image segmentation*: the MR image is segmented based on the signal intensity. A binary mask is generated by setting a threshold to exclude all the pixels with lower than half of the maximum intensity (Figure 2(b)).
3. *Boundary delineation*: the exterior boundary pixels of the binary mask are extracted. The number of points is then reduced by choosing one of every five pixels. This reduced set is used to generate a finite element mesh (Figure 2(c)).
4. *Source and detector position mapping*: as the last step, the source and detector positions are mapped to the coordinates on the mesh manually by placing sixteen lines separated by 22.5 degrees as shown in Figure 2(d).

### 2.3. Phantom Studies

Eight arbitrarily shaped phantoms were constructed to test several different analysis methods. The phantoms were prepared using agarose powder (OmniPur Agarose, Lawrence, KS). Intralipid and Indian ink were added as the optical scattering and absorbing media, respectively. 10 mm inner-diameter thin-walled NMR tubes filled with a dye and Intralipid mixture were inserted at different depths as the inclusions. The optical properties of the homogeneous background were set to *μ*_*a*_ = 0.01 mm^−1^ and *μ*_*s*_′ = 0.6 mm^−1^. The shape of the phantoms and the location of the inclusions are determined from the MR images. For each phantom, inclusions with five different object-to-background contrast (OBC) values were prepared and designated by B0, B2, B4, B6, and B8 (OBC: 1, 2, 4, 6, and 8) that corresponded to *μ*_*a*_ = 0.01, 0.02, 0.04, 0.06, and 0.08 mm^−1^, respectively. The absorption maps were reconstructed individually at each time point. For the DCE-DOT, enhancement images were produced by subtracting preinjection from postinjection reconstructed images. From the set of five different OBC values, B2 through B8 were selected as the postinjection frames, while B0 was assumed as the preinjection (baseline) frame. 

A complete set of data was also acquired using a homogeneous DOT calibration phantom at the end of each case. This was a solid cylindrical homogeneous phantom made of epoxy. The optical properties of the DOT calibration phantom were set to *μ*_*a*_ = 0.006 mm^−1^ and *μ*_*s*_′ = 1.0 mm^−1^. Later, the experimental data (*F*_measured_) was calibrated using the homogeneous phantom measurements (*F*_homo_measured_)

(9) $$F_{\text{calibrated}}=\frac{F_{\text{measured}}}{F_{\text{homo}\_\text{measured}}}\times F_{\text{homo}\_\text{forw}},$$

where *F*_homo_forw_ was calculated by the forward solver. The calibrated data *F*_hete_calibrated_ was then fed into the inverse solver. This step took into account for the data/model mismatch.

### 2.4. Data Analysis

Our first aim is to investigate the effectiveness of the contrast-enhanced DOT compared to regular DOT. A variety of irregularly shaped phantom data allow us to evaluate the modeling mismatch as well as other factors such as optical probe coupling to the tissue surface, source-detector position uncertainty, and errors in delineating medium boundary from the MR images. Our second aim is to investigate whether implementing the decoupling technique or reconstructing absorption images alone instead of together with scattering images can alleviate some of these problems. Above those, our final aim to examine the influence of the structural *a priori* information on the accuracy of DCE-DOT. To address these questions, four methods are used to analyze the experimental data.

**Method No. 1**

Both 64-amplitude and 64-phase data are used to simultaneously reconstruct both absorption and scattering maps. The selection of the ROI for calculating the absorption coefficient is determined by full width half maximum (FWHM) of the inclusion in the DCE-DOT map.

**Method No. 2**

Only 64-amplitude data are used to reconstruct absorption maps. The scattering coefficient of the phantom is assumed to be homogeneous and obtained by fitting a homogeneous absorption and scattering value to both amplitude and phase measurements. The selection of the ROI was determined by FWHM of the inclusion resolved in the DCE-DOT map. Most optical contrast agents introduce only significant absorption change. For this method, the equivalent continuous wave analysis is performed to investigate if continuous wave domain measurements are sufficient for DCE-DOT experiments.

**Method No. 3**

Only 64-amplitude data are used to reconstruct absorption maps. The scattering coefficient of the phantom is also assumed to be homogeneous as in Method no. 2. Afterwards, the DOT reconstruction performed here takes into account the source-detector coupling factors. The selection of the ROI is determined by FWHM of the inclusion resolved in the DCE-DOT map.

**Method No. 4**

MR structural *a priori* information is used in this approach. The ROI is predefined from the MR image. Afterwards, the DOT reconstruction is performed using the structural *a priori* information described earlier in Section 2. The ROI is already determined from MRI as the structural *a priori*.

### 2.5. In Vivo Study

To further validate the hybrid system and our analysis methods, animal studies were carried out. All animal procedures were approved by the Institutional Animal Care and Use Committee at University of California, Irvine. Fischer rats subcutaneously implanted with R3230 adenocarcinoma breast tumor were used for this study. The R3230 cell line is a lactating mammary tumor model that has been previously characterized as a breast cancer model [31]. The cells were allowed to grow until the resulting tumor reached a size of approximately 1 cm, when the DCE-DOT studies were performed.

The FDA-approved optical contrast agent ICG was used in this study. A 785 nm laser was used to perform the DCE-DOT study, which corresponds to the peak absorption of ICG. 32 frames of DOT measurements were obtained throughout each experiment with 16-second temporal resolution. ICG was injected during the 7th frame as a single bolus together with the MR contrast agent Gd-DTPA. The *in vivo* data was also analyzed using two different methods.

**Method No. 1**

Only 64-amplitude data were used to reconstruct absorption maps. The scattering maps were assumed to be homogeneous (*μ*_*s*_′ = 0.8 mm^−1^), which was obtained by fitting the maps to both amplitude and phase data. The selection of the ROI was determined by the FWHM of the inclusion resolved in the peak DCE-DOT map. This was similar to Method no. 2 in the phantom study.

**Method No. 2**

MR structural *a priori* information was used in this approach. The ROI was predefined from the MR image. Then the DOT reconstruction was performed using the structural *a priori* information. This was similar to Method no. 4 in the phantom study.

## 3. Results

Initially, reconstruction results are compared using only Method no. 1 for two different contrast values, B2 and B8, to show the effectiveness of DCE-DOT. While absorption maps are reconstructed individually at each time point for both regular DOT and DCE-DOT, enhancement images are produced by subtracting the B0 image from the B2 and B8 images for all eight DCE-DOT phantom cases. During the comparison, the main criteria are the ability of resolving the inclusion and the reduction of artifacts in the images. After that, different methods such as reconstruction of *μ*_*a*_ only using 64-amplitude data (no. 2), the decoupling method (no. 3) and utilization of MR structural *a priori* information (no. 4) are compared utilizing only the highest contrast, B8 (OBC: 8). Finally, the errors in the recovered mean absorption values are compared for all four methods. This step is repeated for each contrast value for all eight cases. The recovered values as well as the difference between the recovered values and the expected values are also plotted.

### 3.1. Comparison between DCE-DOT and Regular DOT

**Figure 3:**
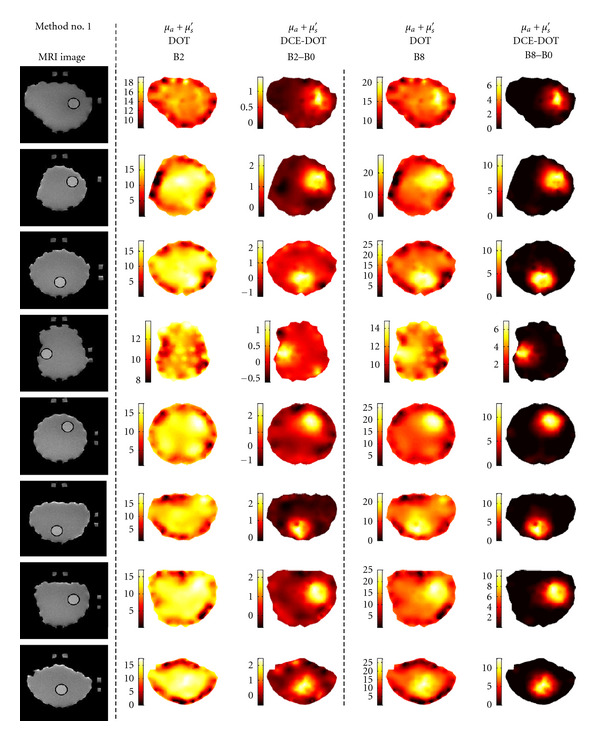
Comparison between regular DOT and DCE-DOT. Column 1: MR images of all the phantoms. Columns 2 and 4: regular endogenous DOT reconstruction for the contrasts B2 and B8, respectively. At B2 contrast, regular DOT cannot resolve the inclusion accurately, and the images are also contaminated by the background noise significantly. Even though all of the inclusions can be located when contrast is high, artifacts are still present in the regular DOT image. Columns 3 and 5: DCE-DOT reconstruction for contrast B2 (B2–B0) and B8 (B8–B2), respectively. The inclusions are clearly identified for both low and high contrast. This shows the superiority of DCE-DOT.

The results are shown in Figure 3. The MR image of each phantom is listed in the first column. Data analysis Method no. 1 is used for the DOT reconstruction, where both phase and amplitude are used to reconstruct both the absorption and scattering maps. The reconstructed regular DOT and DCE-DOT absorption maps for the low contrast, B2 (OBC: 2), are shown in the second and third columns, respectively. Similarly, the reconstructed maps for the high contrast, B8 (OBC: 8), are shown in the fourth and fifth columns. When the contrast is low, regular DOT cannot resolve the inclusions confidently for all cases, and the images are also significantly contaminated by the background artifacts. For instance, the inclusions can be resolved in Cases 3, 5, and 7, even though many artifacts are present. At the same time, they cannot even be localized in all the other cases (Figure 3-column two). When the contrast increases to eight, however, inclusions are clearly identified for all cases (Figure 3-column four). If DCE-DOT is utilized, on the other hand, they are clearly resolved for all of the cases, even if the OBC is as small as two (Figure 3-column three). As expected, the quality improves further when the contrast increases to eight (Figure 3-column five). 

These initial results demonstrate the superiority of the DCE-DOT method, that is the reconstructed images significantly improved from columns two to three. The artifacts in the background are also significantly diminished. The results show that it is very difficult for regular DOT to resolve small objects in an arbitrarily shaped medium. The model might fail due to the insensitivity of small perturbations in a heterogeneous medium. However, DOT can be very sensitive to the increase of absorption in the inclusion (e.g., administration of contrast agent), and, hence, it can be easily localized with DCE-DOT.

### 3.2. Comparison between Different DCE-DOT Reconstruction Methods

**Figure 4:**
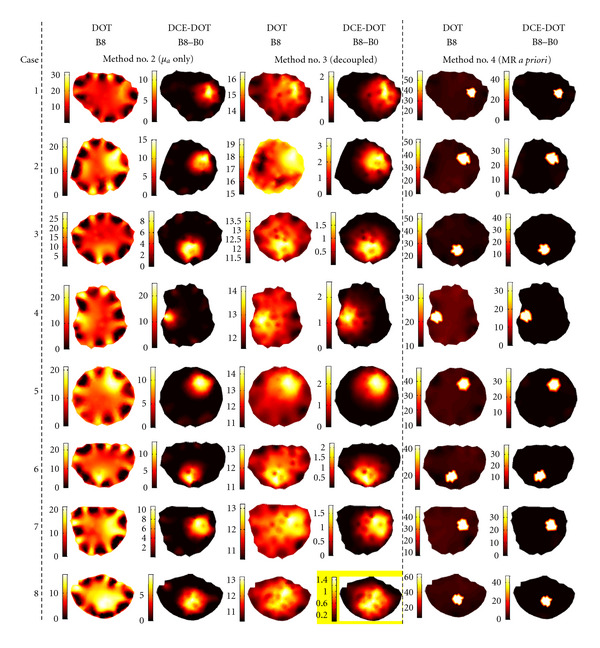
Comparison among different methods of reconstruction for the absorption coefficient, *μ*_*a*_ (m^−1^), please note that unit of m^−1^ is used for display purposes. The first and second columns present the reconstructed regular DOT and DCE-DOT absorption maps for B8 using analysis Method no. 2 (reconstructing *μ*_*a*_ only). The third and fourth columns show the reconstructed maps using analysis Method no. 3 (taking the source-detector coupling factor into account), while the fifth and sixth columns are for the reconstructed maps using analysis Method no. 4 (MR *a priori* information is used). When the source-detector coupling factor is considered, the hypersensitivity at the source-detector positions due to coupling errors is reduced. Furthermore, when MRI *a priori* information is utilized, the inclusion can be clearly identified without background artifacts.

In this section, we intend to address the effectiveness of other methods described in Section 2.4. The results are shown in Figure 4. Respectively, the first and second columns are the reconstructed regular DOT and DCE-DOT absorption maps for the highest contrast, B8, using analysis Method no. 2 (reconstructing *μ*_*a*_ only). Similarly, the third and fourth columns present the reconstructed maps using analysis Method no. 3 (accounting for the coupling factor), while the fifth and sixth columns show the reconstructed maps using analysis Method no. 4 (with MR *a priori* information).

#### 3.2.1. Reconstruction of Both *μ*_*a*_ and *μ*_*s*_′ versus Reconstruction of *μ*_*a*_ Only

The results of the reconstruction of both *μ*_*a*_ and *μ*_*s*_′ are presented in the fourth and fifth columns in Figure 3 (Section 3.1), while the results of the reconstruction of the *μ*_*a*_-only approach is shown in the first and second columns of Figure 4. For the regular DOT reconstruction, the inclusions are resolved with fewer artifacts when both *μ*_*a*_ and *μ*_*s*_′ are simultaneously reconstructed. Apparently, the error in the scattering coefficient affects the reconstructed results if *μ*_*a*_ is reconstructed by itself. For instance, the inclusions are not visible for Cases 1, 3, and 4 in Figure 4-column one.

This demonstrates the limitation of a continuous wave technique, which cannot effectively separate scattering and the absorption coefficients. These effects are expected to be more severe for *in vivo* situations, where tissue is more heterogeneous. Despite this issue, the DCE-DOT images for both methods, *μ*_*a*_ and *μ*_*s*_′ or *μ*_*a*_ only, clearly resolve the inclusions in all of the cases. The enhancement absorption images produced in DCE-DOT are only sensitive to the absorption variation due to the contrast agent. Hence, even though there are modeling and experimental errors in each frame for the *μ*_*a*_-only reconstruction, the resulting enhancement maps consistently resolve each inclusion for each case.

#### 3.2.2. Reconstruction with versus without Source-Detector Decoupling

The reconstruction results with and without source-detector coupling are shown in Figure 4. As expected, the hypersensitivity at the source-detector positions is significantly reduced if the coupling factor is considered. This validates the importance of taking into account the coupling factor during regular DOT studies. Similar to the previous method, the recovered images using DCE-DOT using both methods are comparably good. Meanwhile, it should be noted that the contrast is reduced when the source-detector coupling factor is considered. Fang et al. also reported this phenomenon previously [7]. Indeed, the coupling factor decreases the object-to-background contrast. In summary, the source-detector decoupling method improves the regular DOT reconstruction results but decreases the recovered contrast for both regular DOT and DCE-DOT.

#### 3.2.3. Reconstruction with versus without MR A Priori Information

The reconstruction results utilizing MR *a priori* information are shown in the fifth and sixth columns in Figure 4. Significant improvement is obtained when MR *a priori* information is utilized. For regular DOT, the recovered absorption for the inclusion is strongly enhanced by the anatomical constraint. As expected, the DCE-MRI results shown in the sixth column are clearly without artifacts in the background. These results conclude that the absorption inclusion can be consistently recovered when the MRI anatomical information is used.

### 3.3. Comparison among Different DCE-DOT Analysis Methods

**Figure 5:**
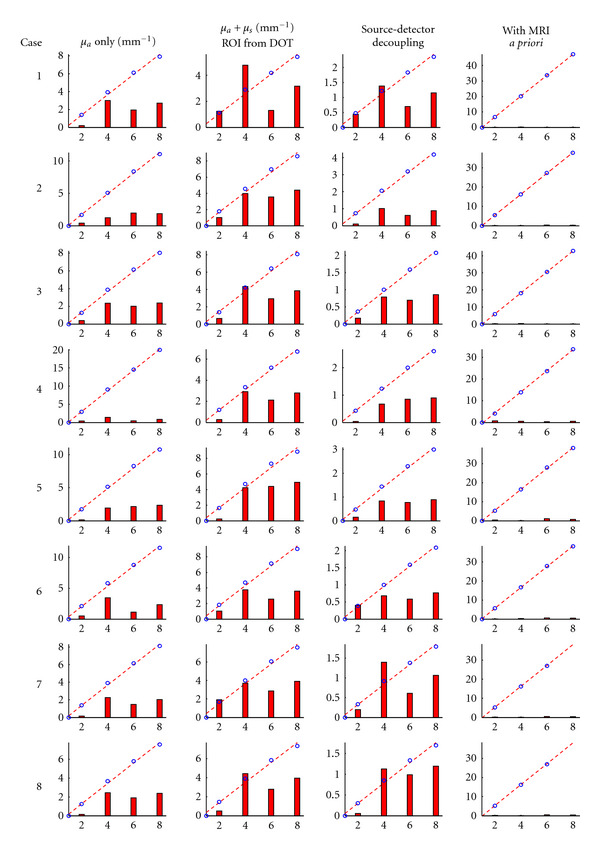
Plots showing the average reconstructed absorption increase in the region of interest at all five concentrations. Please note that unit of m^−1^ is used for display purposes. The blue circles are the actual data, whereas the red dashed line is a linear fit to this data. The red bar graphs represent the deviation between the data and the linear fit. This deviation was obtained by taking the absolute value of the difference between the data and the linear fit, normalizing it with respect to the maximum value of the data (blue circles), and then multiplying by a constant to elucidate the deviation. Each column of graphs contains data from one of the reconstruction methods, while each row contains the graphs from a given case.

Recovering accurate enhancement kinetics of a contrast agent using DCE-DOT necessitates a linear relationship between the signal variation and the concentration of agent. Accordingly, we test the linearity of the recovered absorption coefficient of the inclusion for each case at the final step. For each analysis method described in Section 2.4 (Table 1) a linear fit was performed as shown in Figure 5. The red dashed line represents the linear fit to the data points in each graph. The red bar graph represents the deviation of the data from the linear fit. As seen from the figure, there is a comparatively large deviation from the linear fit without MR spatial *a priori,* regardless of the optical reconstruction method. In contrast, this nonlinearity is remedied by using MR *a priori* information, as shown in the fourth column in Figure 5, where the difference between the fit and the actual data is much smaller. 

**Figure 6:**
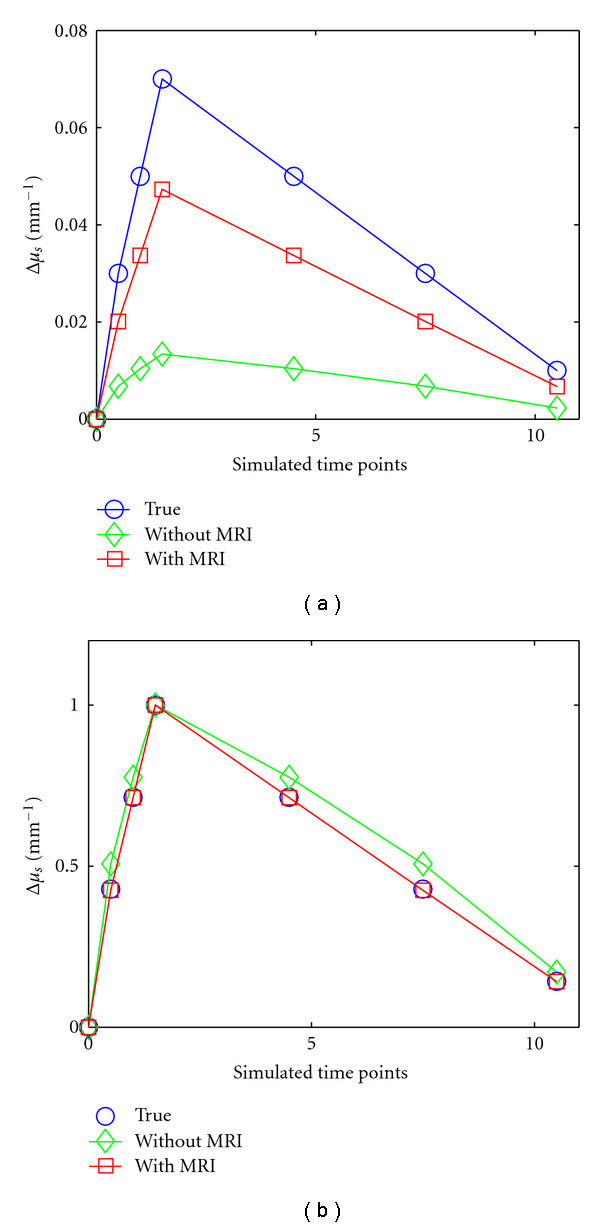
Enhancement kinetics curve analysis of the first case. The solid blue line represents the true value of the inclusion's optical properties, while the red and green lines represent the recovered values with and without MR *a priori* information. Each point on the graph is taken from a different concentration phantom case (B0, B2, etc.) and is organized in this way to simulate a synthetic enhancement curve.

To show the effect of this nonlinearity on the recovered enhancement curve, Case no. 1 data was used for further analysis. For this purpose, a synthetic enhancement kinetics curve is created using all five data points. The true curve is shown as the solid blue line in Figure 4. The recovered enhancement kinetics with and without MR *a priori* information (Methods no. 3 and no. 5) are shown as the green and red lines, respectively. The recovered absorption increase is more accurate when MRI information is utilized (Figure 6(a)). In general, the normalized kinetics is often used in DCE analysis. Accordingly, the normalized kinetics curve is plotted in Figure 6(b). The recovered enhancement curve matches to the true synthetic curve only when the optical reconstruction is guided and constrained using MR information. These results confirm that the best way to analyze DCE-DOT data is to utilize structural *a priori* information.

### 3.4. In Vivo Study

**Figure 7:**
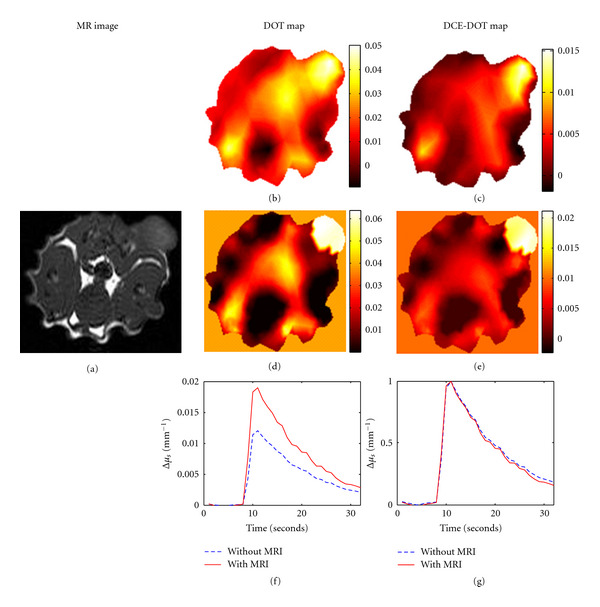
Results from an *in vivo* study using Fischer rats for both regular and dynamic contrast enhancement DOT. (a) shows the T1 image of the rat at the slice that DOT data were acquired. (b) and (d) show the peak reconstructed DOT maps without and with MRI *a priori* information, respectively. (c) and (e) show the DCE-DOT differential maps without and with *a priori *information (i.e., the baseline map is subtracted from the peak absorption map). Finally, (f) shows the dynamic enhancement curves using the recovered absorption coefficient in the ROI. (g) displays the normalized DCE curves with respect to the maximum value.

The anatomic T1-weighted MR image of the rat is shown in Figure 7(a). The tumor region is indicated with the yellow circle. The size of the tumor is approximately 1.5 cm. in diameter. First, DOT and DCE-DOT are compared for their ability to resolve the tumor. For this purpose the reconstructed peak DOT absorption map is compared with the difference image (DCE-DOT) obtained by subtracting the baseline image from the peak DOT absorption map. This step is also repeated without and with MR *a priori* information. The peak reconstructed absorption maps without and with MRI anatomical information are shown in Figures 7(a) and 7(c), respectively. The DCE-DOT difference maps at the peak absorption without and with MRI are also shown in Figures 7(b) and 7(d). The tumor region is visible and clearly shows higher absorption in DOT images. Still, there are several hot spots present in the image as well. However, the artifacts are significantly suppressed on DCE-DOT differential maps. Meanwhile, both regular DOT and DCE-DOT differential maps are improved when MRI anatomical *a priori* information of the tumor location is used for the DOT reconstruction. 

In addition to this, the ICG enhancement kinetics curve is also produced with and without MR *a priori*. Figure 7(e) shows the kinetics of the absolute absorption change, while Figure 7(f) shows the normalized kinetics with respect to the maximum value. The increase in absorption coefficients is considerably underestimated without MR *a priori* information. Furthermore, the normalized kinetics is separated during the wash-out phase. This is in agreement with our phantom results, in which the enhancement kinetics deviated without MRI *a priori *information. In conclusion, the utilization of MRI *a priori* information for *in vivo* DCE-DOT study is demonstrated to be functional.

## 4. Conclusion and Discussion

In this paper, we investigate various analysis methods for DCE-DOT. The significance of this study is threefold. First, the DCE-DOT data is obtained using a hybrid MRI-DOT system. Therefore, the utilization of anatomical *a priori* information for DCE-DOT can be investigated. This is the first time anatomical *a priori* information from another high spatial resolution imaging modality is applied in DCE-DOT analysis. Second, various DOT reconstruction methods are investigated in realistic arbitrarily shaped agar phantoms. Regular shaped phantoms such as cylindered or slab phantoms are used in most previous phantom studies. However, the shape of the phantom affects the reconstruction significantly due to the modeling mismatch, thus eight phantoms are used in our study to have a wide range of geometries. The quantitative accuracy of the recovered DCE-DOT enhancement curves is compared among several analysis methods. We concluded that DCE-DOT with MRI *a priori* information provides the best enhancement images and most accurate enhancement kinetics. Finally, we investigated an *in vivo* case. *In vivo* results agree with the phantom studies. The difference in the enhancement kinetics obtained without and with MR structural *a priori* was not drastic as in the case of phantom studies, presumably due to the close proximity of the tumor to the boundary.

In this study, we showed that the high-absorption area enhanced by exogenous contrast is consistently localized by the DCE-DOT technique without many background artifacts. The results suggest that DCE-DOT can reliably resolve a lesion. However, it is worth noting that DCE-DOT and regular DOT provide essentially different physiological parameters. For example, multiwavelength DOT provides total hemoglobin concentration, oxygen saturation, and other optical markers for cancer imaging. This information cannot be obtained by DCE-DOT, though higher enhancement might correlate well with total hemoglobin contents. On the other hand, DCE-DOT has the potential to provide additional tumor information about morphological vascular differences from the enhancement kinetics [15, 16]. Due to this reason, it is important to obtain an accurate kinetics curve. Unlike DCE-MRI, DCE-DOT does not have high spatial resolution. As a result, the recovered enhancement kinetics depends on the analysis technique and choice of the ROI. We demonstrated in this paper that accurate ICG kinetics can only be recovered with MR spatial *a priori* information.

ICG pharmacokinetics have been investigated previously and found to be different between healthy and diseased tissues [16]. However, the focus of this paper is to evaluate the best way to acquire accurate ICG kinetics. Thus, pharmacokinetic analysis is not performed. As the next step, more *in vivo* data are being acquired to evaluate if ICG kinetics can be used for tumor diagnosis and therapy monitoring.

## Figures and Tables

**Table 1:** The summary of five analysis methods for phantom studies.

Method		No. 1	No. 2	No. 3	No. 4
64 A + Φ	64 amplitude + 64 phase	64 A + Φ	64 A	64 A	64 A
64 A	64 amplitude				
*μ*_a_ + *μ*_*s*_′	both *μ*_a_ and *μ*_*s*_′ reconstructed	*μ*_*a*_ + *μ*_*s*_′	*μ*_*a*_	*μ*_*a*_	*μ*_*a*_
*μ*_*a*_	only *μ*_*a*_ is reconstructed				
Decoup	Decoupling technique is used	—	—	Decoup	—
DOT	ROI is obtained from DOT	DOT	DOT	DOT	MRI
MR	ROI is obtained from MRI				
MR priori	MR structural *a priori *is used	—	—	—	MR *a priori *
